# The Association Between Body Fat Index and Gestational Diabetes Mellitus: A Prospective Cohort Study

**DOI:** 10.7759/cureus.39615

**Published:** 2023-05-28

**Authors:** Sawanya Benchahong, Prasert Sunsaneevithayakul, Dittakarn Boriboonhirunsarn

**Affiliations:** 1 Obstetrics and Gynecology, Faculty of Medicine Siriraj Hospital, Mahidol University, Bangkok, THA

**Keywords:** visceral adipose tissue, subcutaneous adipose tissue, body mass index, gestational diabetes, body fat index

## Abstract

Background: Body mass index (BMI) has commonly been used to evaluate the risk of gestational diabetes mellitus (GDM), but BMI does not always represent body fat mass distribution. Body fat index (BFI), which includes the measurement of subcutaneous adipose tissue (SAT) and visceral adipose tissue (VAT), has been suggested to be a better predictor for GDM than BMI.

Objective: The objective of this study is to compare the risk of GDM among pregnant females with BFI of >0.5 and ≤0.5.

Methods: Maternal abdominal subcutaneous adipose tissue (SAT) and visceral adipose tissue (VAT) thickness were measured by ultrasonography before 14 weeks of gestation, and BFI was calculated (VAT×SAT/height). The study group was 160 females with BFI of >0.5, and the comparison group was 80 females with BFI of ≤0.5. All females received GDM screening during the first antenatal visit and at 24-28 weeks of gestation. The rate of GDM was compared between the two groups. The correlation between BFI and BMI and their diagnostic ability for GDM were evaluated. Logistic regression analysis was performed to determine the independent associated factors for GDM.

Results: Females with BFI of >0.5 were significantly older (p=0.033) and had higher body mass index (BMI) (p<0.001) and were more likely to be overweight or obese (p<0.001). BFI correlated well with BMI (correlation coefficient of 0.736, p<0.001). GDM was significantly more common in females with BFI of >0.5 (24.4% versus 11.3%, p=0.017). The diagnostic ability for GDM between BFI and BMI was similar (areas under receiver operating characteristic {ROC} curves of 0.641 and 0.646, respectively). Significant independent risk factors for GDM were a BFI of >0.5 and a BMI of ≥25 kg/m^2^ (adjusted odds ratio {OR}, 3.8; 95% confidence interval {CI}, 1.5-9.2), age of ≥30 years (adjusted OR, 2.8; 95% CI, 1.2-6.4), and family history of diabetes mellitus (DM) (adjusted OR, 4.0; 95% CI, 1.9-8.3).

Conclusion: Females with BFI of >0.5 were significantly more likely to have GDM. The diagnostic ability of BFI and BMI for GDM was comparable. Females with BFI of >0.5 and BMI of ≥25 kg/m^2^ have an increased risk for GDM.

## Introduction

Gestational diabetes (GDM) is a common condition with varying prevalence according to population, diagnostic criteria, ethnicity, and geographic location. There is a worldwide increase in GDM, probably due to an increase in maternal obesity [1,2]. GDM is often associated with various maternal and fetal complications such as preeclampsia, large-for-gestational-age infants, fetal macrosomia, shoulder dystocia, increase in cesarean delivery rate, respiratory distress syndrome, and neonatal hypoglycemia. Thus, the proper management of GDM through lifestyle modification, diet control, and medical treatment may lead to a decrease in such complications [1-3].

Body mass index (BMI) has commonly been used to evaluate the risk of obesity-related pregnancy complications, including GDM. However, BMI does not distinguish between bone, muscle, and fat mass; that is, it might not reflect the fat mass distribution of each pregnant female and might not be good enough to predict the risk of GDM. On the other hand, maternal central obesity would be more significant and might better reflect fat distribution that should be used as an important risk for GDM [4-7]. Many measurements for maternal central obesity have been reported, including waist circumference [4], waist-to-hip ratio [6], and body fat composition [8-10]. Several studies have reported the associations between abdominal subcutaneous fat thickness (ASFT) and abdominal visceral fat thickness (AVFT) measured by ultrasonography during the first and second trimesters and the development of GDM [11-22]. A recent systematic review reported that maternal central obesity was directly associated with the risk of developing GDM (adjusted odds ratio {OR}: 2.76) and visceral adipose tissue (VAT) had the highest odds ratio for GDM; that is, it might play an important role in developing GDM [5].

Body fat index (BFI), which is calculated by SAT (mm)×VAT (mm)/height (cm), has been proposed as another measure for maternal central obesity. A BFI of >0.5 has been shown to be associated with GDM and hypertensive disorders in pregnancy. In addition, it was also suggested that BFI was a better predictor for both conditions than BMI [23]. As the ultrasound measurement of subcutaneous adipose tissue (SAT) and VAT has been shown to be practical and easy, their use to predict GDM, either alone or in combinations, could be cost-effective [24].

In Thailand, BMI is commonly used for GDM risk stratification, while maternal central obesity is not. Information on its association with GDM is still lacking, especially for BFI. Therefore, the objective of this study was to evaluate if BFI during the first trimester can predict GDM, by comparing GDM diagnosis between pregnant females with BFI of ≤0.5 and >0.5. In addition, the diagnostic ability of BFI and BMI, as well as the relationship between BFI and BMI, was evaluated. The results would provide some new perspectives on GDM risk stratification that could be applied into clinical practice.

This article was previously presented as a meeting poster at the 2022 International Congress of Diabetes and Metabolism on October 6-8, 2022, in Seoul, South Korea.

## Materials and methods

After approval from the Siriraj Institutional Review Board (certificate of approval {COA} number: Si 884/2020), a prospective cohort study was conducted at the Department of Obstetrics and Gynecology of the Faculty of Medicine Siriraj Hospital in 2021. Eligible females were those with a singleton pregnancy, who were >18 years old, started antenatal care before 14 weeks of gestation, and received GDM screening according to institutional guidelines. Pregnant females who had prepregnancy diabetes, intrauterine fetal death in the current pregnancy, upper abdominal surgical scar, and hepatomegaly and could not communicate in Thai were excluded. The sample size was estimated from a pilot study that showed that the ratio of females with BFI of >0.5 and ≤0.5 was 2:1 and the rate of GDM was 25% and 10%, respectively. At 95% significance level and 80% power, at least 156 females with BFI of >0.5 and 78 with BFI of ≤0.5 were required (2:1 ratio).

After written informed consent, all participating females were scheduled for an ultrasonographic measurement of abdominal adipose tissue before 14 weeks of gestation. In the assessment of adipose tissue, pregnant females were placed in a supine position with their bodies in a straight line, their backs being adjacent to the examination bed. A longitudinal scan of the maternal abdomen was obtained, applying the minimum pressure with the ultrasound transducer on the abdomen. The ultrasound probe was placed perpendicular to the skin at the midline epigastrium.

**Figure 1:**
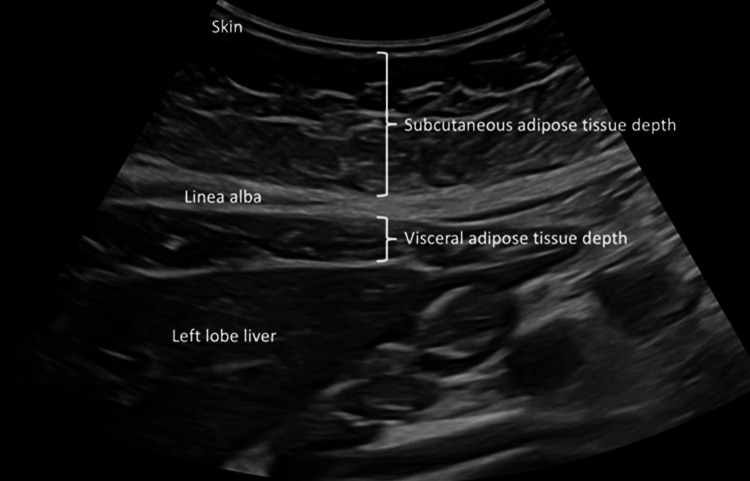
Ultrasonographic measurement of subcutaneous and visceral adipose tissue thickness Image credit: Sawanya Benchahong

Ultrasonographic measurements are displayed in Figure 1 [23]. Subcutaneous adipose tissue (SAT) thickness was measured as the minimum vertical distance from the skin line to the upper edge of the linea alba, immediately caudal to the xiphoidal tip. The maximum visceral adipose tissue (VAT) thickness was measured on the same image from the lower edge of the linea alba to the anterior surface of the liver. The measurement was performed at the end of the normal inspiration act to avoid the distortion of the abdominal cavity. The caliper measurements were always kept perpendicular to the surface. The measurements were performed by a single obstetrician, well-trained by experienced staff in the maternal and fetal medicine unit, who was blinded for GDM screening results. Intraclass coefficients were 0.97 and 0.98 for the intra- and inter-observer reliability evaluation of the measurements. Two measurements were taken, and mean values were used in further calculations. Body fat index (BFI) was calculated using the following formula: BFI=[VAT (mm)×SAT (mm)]/height (cm) [23]. Those with BFI of >0.5 and ≤0.5 were assigned to the study and comparison groups, respectively.

According to institutional guidelines, all pregnant females received GDM screening during their first antenatal care visit and repeat at 24-28 weeks of gestation. A 50 g glucose challenge test was used as a screening test at 140 mg/dL cutoff value, and a 100 g oral glucose tolerance test was used as a diagnostic test by the Carpenter and Coustan criteria. GDM diagnosed from first screening was considered as early-onset GDM, while those diagnosed during 24-28 weeks of gestation were considered as late-onset GDM. All females received standard antenatal care according to institutional protocol. Those who were diagnosed with GDM were offered counseling and nutritional therapy. Insulin therapy was initiated in females with poor glycemic control. Fasting plasma glucose and/or two-hour postprandial plasma glucose was used for follow-up with 95 mg/dL and 140 mg/dL as glycemic control targets, respectively. Data were collected and recorded, including baseline and obstetric characteristics, GDM risk factors, ultrasonographic evaluation of adipose tissue (VAT, SAT, and BFI), GDM screening results, diagnosis, and management.

Descriptive statistics, including mean, standard deviation, number, and percentage, were used to describe various characteristics as appropriate. Student's t test and chi-square test were used for the comparison of various characteristics and outcomes between the study and comparison groups as appropriate. The correlation between BFI and BMI was evaluated, and correlation coefficient was estimated. Receiver operating characteristic (ROC) curves and areas under the curved were used to determine the diagnostic ability of BFI and BMI for GDM. The risk of GDM according to BFI and BMI, individually and in combination, was evaluated, and relative risks (RR) and 95% confidence intervals (CI) were estimated. Logistic regression analysis was used to determine independent risk factors for GDM, adjusting for potential confounders. A p value of <0.05 was considered statistically significant.

## Results

A total of 240 pregnant females were included in this study, 80 had BFI of <0.5 (comparison group), and the other 160 had BFI of >0.5 (study group). Table 1 shows the comparison of baseline characteristics between the two groups. Females with BFI of >0.5 were significantly older (p=0.033) and had higher BMI (p<0.001). Regarding GDM risks, females with BFI of >0.5 were more likely to be overweight or obese (p<0.001). Other characteristics were comparable.

**Table 1:** Comparison of baseline characteristics between the two groups BFI, body fat index; SD, standard deviation; BMI, body mass index; GDM, gestational diabetes mellitus; DM, diabetes mellitus

Characteristics	BFI of ≤0.5 (N=80)	BFI of >0.5 (N=160)	P value
	Mean±SD	Mean±SD	
Age (years)	29.7±5.4	31.1±4.5	0.033
BMI (kg/m^2^)	19.8±2.3	25.5±4.85	<0.001
	N (%)	N (%)	
Nulliparity	42 (52.5%)	83 (51.9%)	0.927
GDM risks			
Age of ≥30 years	46 (57.5%)	103 (64.4%)	0.301
Family history of DM	16 (20.0%)	38 (23.8%)	0.512
BMI of ≥25 kg/m^2^	0 (0.0%)	79 (49.4%)	<0.001
Previous GDM	1 (1.3%)	4 (2.5%)	0.667
Previous macrosomia	2 (2.5%)	0 (0%)	0.110
Chronic hypertension	2 (2.5%)	5 (3.1%)	1.000

Table 2 shows ultrasonographic measurements between the two groups. Measurements were performed at approximately 12 weeks of gestation in both groups. Every measurement was significantly higher in females with BFI of >0.5, including SAT, VAT, and BFI (p<0.001).

**Table 2:** Comparison of ultrasonographic measurement characteristics between the two groups BFI, body fat index; SD, standard deviation; GA, gestational age; SAT, subcutaneous adipose tissue; VAT, visceral adipose tissue

Characteristics	BFI of ≤0.5 (N=80)	BFI of >0.5 (N=160)	P value
	Mean±SD	Mean±SD	
GA at ultrasound	12.18±0.57	12.18±0.62	0.940
SAT (mm)	9.02±3.00	17.07±5.39	<0.001
VAT (mm)	5.25±1.93	11.67±3.98	<0.001
BFI	0.30±0.13	1.30±0.76	<0.001

**Figure 2:**
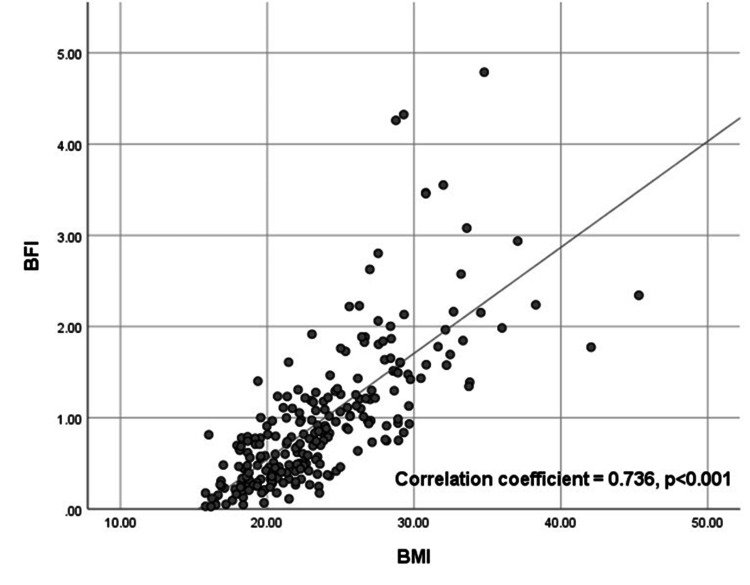
Scatter plot of BFI and BMI Image credit: Dittakarn Boriboonhirunsarn BFI, body fat index; BMI, body mass index

The correlation between BMI and BFI is demonstrated in a scatter plot diagram as shown in Figure 2. A significant positive correlation was observed with a correlation coefficient of 0.736 (p<0.001). A comparison of the BFI and BMI groups is shown in Table 3, and a significant association was observed (p<0.001). No females with BFI of ≤0.5 were overweight or obese, while 49.4% were among those with BFI of >0.5. Among females whose BFI was >0.5, 46.9% had normal BMI, and 3.8% were underweight.

**Table 3:** Comparison of BMI categories between the two groups BFI, body fat index; BMI, body mass index

	BFI of ≤0.5 (N=80)	BFI of >0.5 (N=160)	P value
BMI category			
Underweight (<18.5 kg/m^2^)	26 (32.5%)	6 (3.8%)	<0.001
Normal (18.5-24.9 kg/m^2^)	54 (67.5%)	75 (46.9%)	
Overweight (≥25 kg/m^2^)	0 (0.0%)	79 (49.4%)	

Table 4 shows the comparison of GDM diagnosis between the two groups. The gestational age (GA) at the first and second screenings was comparable at eight and 26 weeks of gestation. GDM was diagnosed in 24.4% among females with BFI of >0.5, while it was 11.3% among those with BFI of ≤0.5 (p=0.017). In addition, both early-onset and late-onset GDM were more common in females with BFI of >0.5 but without statistical significance (18.1% versus 10% {p=0.100} and 7.6% versus 1.4% {p=0.101}, respectively).

**Table 4:** Comparison of GDM diagnosis between the two groups BFI, body fat index; SD, standard deviation; GDM, gestational diabetes mellitus; GA, gestational age

Characteristics	BFI of ≤0.5 (N=80)	BFI of >0.5 (N=160)	P value
	Mean±SD	Mean±SD	
GA at first screening (weeks)	8.3±2.4	8.7±2.5	0.219
GA at second screening (weeks)	26.0±1.8	26.2±1.9	0.533
GA at diagnosis (weeks)	9.4±7.7	12.8±8.9	0.296
	N (%)	N (%)	
GDM diagnosis	9 (11.3%)	39 (24.4%)	0.017
Early-onset GDM	8 (10.0%)	29 (18.1%)	0.100
Late-onset GDM	1/72 (1.4%)	10/131 (7.6%)	0.101
Insulin therapy	1/9 (11.1%)	4/39 (10.3%)	1.000

**Figure 3:**
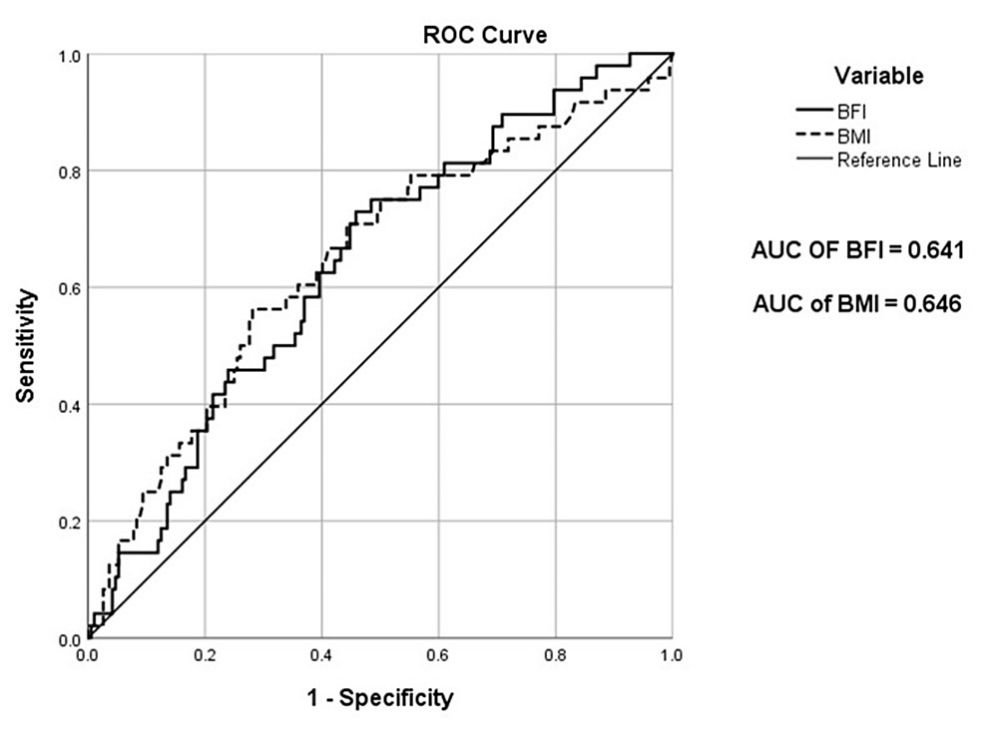
Receiver operating characteristic (ROC) curve of BFI and BMI in GDM diagnosis Image credit: Dittakarn Boriboonhirunsarn BFI, body fat index; BMI, body mass index; GDM, gestational diabetes mellitus; AUC, area under the curve

An ROC curve was used to evaluate the diagnostic ability of GDM between BFI and BMI as shown in Figure 3. The curves were similar for both BFI and BMI with comparable areas under ROC curves of 0.641 and 0.646, respectively.

The risk of GDM according to BFI and BMI was evaluated, and the results are shown in Table 5. Significant increase in GDM risk was observed in females with BFI of >0.5 and BMI of ≥25 kg/m^2^ (RR: 2.2 and 95% CI: 1.1-4.2 and RR: 2.4 and 95% CI: 1.5-4.0, respectively). In terms of diagnostic values for GDM, a BFI of >0.5 had a sensitivity of 81.3% and a specificity of 37%, while a BMI of ≥25 kg/m^2^ had a sensitivity of 54.2% and a specificity of 72.4%. When BFI and BMI were considered together, females with both BFI of >0.5 and BMI of ≥25 kg/m^2^ significantly increased the risk of GDM (RR, 3.0; 95% CI, 1.5-5.9), while those with BFI of >0.5 only did not significantly increase the risk.

**Table 5:** Risk of GDM according to BFI and BMI For a BFI of >0.5, sensitivity=81.3%, and specificity=37%. For a BMI of ≥25 kg/m^2^, sensitivity=54.2%, and specificity=72.4% BFI, body fat index; RR, relative risk; BMI, body mass index; GDM, gestational diabetes mellitus; CI, confidence interval

	GDM	No GDM	RR (95% CI)	P value
BFI				0.017
≤0.5 (N=80)	9 (11.3%)	71 (88.7%)	1.0	
>0.5 (N=160)	39 (24.4%)	121 (75.6%)	2.2 (1.1-4.2)	
BMI				<0.001
<25 kg/m^2^ (N=161)	22 (13.7%)	139 (67.1%)	1.0	
≥25 kg/m^2^ (N=79)	26 (32.9%)	53 (67.1%)	2.4 (1.5-4.0)	
BFI and BMI				0.002
BFI of ≤0.5 and BMI of <25 kg/m^2^ (N=80)	9 (11.3%)	71 (88.2%)	1.0	
BFI of >0.5 only (N=81)	13 (16%)	68 (84%)	1.4 (0.6-3.1)	0.375
BFI of >0.5 and BMI of ≥25 kg/m^2^ (N=79)	26 (32.9%)	53 (67.1%)	3.0 (1.5-5.9)	<0.001

Logistic regression analysis was performed to determine the independent risk factors for GDM, and the results are shown in Table 6. Significant independent risk factors were both a BFI of >0.5 and a BMI of ≥25 kg/m^2^ (adjusted OR, 3.8; 95% CI, 1.5-9.2), an age of ≥30 years (adjusted OR, 2.8; 95% CI, 1.2-6.4), and a family history of diabetes mellitus (DM) (adjusted OR, 4.0; 95% CI, 1.9-8.3).

**Table 6:** Logistic regression analysis to determine the independent risk for GDM BFI, body fat index; OR, odds ratio; CI, confidence interval; BMI, body mass index; DM, diabetes mellitus; GDM, gestational diabetes mellitus

	Adjusted OR (95% CI)	P value
BFI of ≤0.5 and BMI of <25 kg/m^2^	1.0	
BFI of >0.5 only	1.4 (0.6-3.7)	0.467
BFI of >0.5 and BMI of ≥25 kg/m^2^	3.8 (1.5-9.2)	0.003
Age of ≥30 years	2.8 (1.2-6.4)	0.013
Family history of DM	4.0 (1.9-8.3)	<0.001
Multiparous	0.6 (0.3-1.3)	0.224

## Discussion

The results of this study showed that GDM was significantly more common in females with BFI of >0.5 (24.4% versus 11.3%, p=0.017) with similar diagnostic ability for BMI and BFI. A BFI of >0.5 and a BMI of ≥25 kg/m^2 ^independently increased the risk of GDM with an adjusted OR of 3.8 (95% CI: 1.5-9.2).

Although BMI is a generally accepted measurement of body fat, it does not provide details on either the quantity or the quality of body fat, as well as its distribution, that is, subcutaneous adipose tissue (SAT) or visceral adipose tissue (VAT). Previous studies showed that maternal central obesity would be more significant and might better reflect fat distribution that could be used as an important risk for GDM [4-7]. A recent systematic review reported that maternal central obesity, especially visceral adipose tissue (VAT), was directly associated with the risk of developing GDM, probably by increasing insulin resistance and chronic inflammatory response [5,25,26]. Several previous studies have reported the associations between GDM development and SAT and VAT measured by ultrasonography during the first [11-13,17-21,25] and second trimesters [14-16,22]. Recently, body fat index (BFI) has been proposed as another measure for maternal central obesity by combining together SAT, VAT, and height. A BFI of >0.5 has been shown to be associated with GDM and hypertensive disorders in pregnancy, and it could be a better predictor for both conditions than BMI [23]. The results of this study showed that a BFI of >0.5 significantly increased the risk of GDM compared to those with BFI of ≤0.5 (24.4% versus 11.3%, p=0.017), which was in concordance with a previous study [23].

The results of this study also showed that BFI correlated well with BMI with a correlation coefficient of 0.736. Similar results were observed from a previous study [23]. It should also be noted that, in the study samples, there were no overweight or obese females among those with BFI of ≤0.5 and among those with BFI of >0.5; almost half had normal BMI. This suggests that BMI might not always reflect all the body fat as noted from previous studies [4-7].

The results also demonstrated that the diagnostic abilities of BFI and BMI for GDM were comparable, with similar areas under the ROC curves. At these cutoff values, BFI provided higher sensitivity but lower specificity compared to BMI. This was in contrast to a previous study that reported that a BFI of >0.5 was statistically superior to a BMI of >25 or 30 kg/m^2^ as a predictor of GDM [23]. On the other hand, one previous study reported that BMI was a more useful predictor than the other anthropometric tools, including SAT and VAT [27].

Regarding other measurements of central obesity, recent studies reported that VAT may be a more sensitive predictor of GDM than BMI [11,22]. Another study reported that the ultrasonographic measurement of VAT in early pregnancy had a twofold increase in sensitivity to almost 90% for identifying GDM compared to the use of selective screening protocol [28]. SAT has also been reported to predict GDM with a sensitivity of 72%-76.6% and a specificity of 60%-91.7% [12,14].

When both BFI and BMI were considered together, a significant increased risk of GDM was observed in females with BFI of >0.5 and BMI of ≥25 kg/m^2^ with a relative risk of 3.0. And after potential confounders were taken into account, the adjusted OR was 3.8. It should be noted that a BFI of >0.5 only did not significantly increase the risk of GDM. The use of both indices together could help in increasing the ability to diagnose GDM. Therefore, BFI could be useful as an additional factor to BMI to stratify the risk of GDM.

Varying results on central obesity and GDM between studies might be due to differences in population characteristic, GDM risks, and screening and diagnostic protocol used between studies. In addition, there were also differences in central obesity evaluations (SAT, VAT, BFI, and BMI), as well as the timing of measurements (first or second trimester). Therefore, it might not be easy to validly and reliably compare these results between studies and make conclusion on its usefulness. Body fat index (BFI) is relatively new to obstetric practice, and there is still limited data on its use. There is only one study on its potential use for the prediction of GDM and hypertensive disorders in pregnancy, and the measurement was performed during 18-24 weeks of gestation [23]. The current study is the first to evaluate the use of BFI measured during the first trimester and the risk of GDM. Although the information is still limited, this study could add some perspectives on the potential use of BFI in the future.

The strengths of this study may include that all the measurements were performed by a single, well-trained obstetrician, and the mean of two measurements was used in the calculations and analysis that should help minimize measurement errors. Moreover, all females received uniform GDM screening and diagnosis according to institutional guidelines. However, some limitations should be mentioned. The study was conducted in a single tertiary care university hospital, so the generalization of the results might be limited. The samples might be too few for further subgroup analysis.

As the ultrasonographic measurement of maternal central obesity, including SAT and VAT, is relatively easy to perform without intensive training and needs little expertise, these measurements can be introduced to general practitioners for the evaluation of central obesity. The use of various maternal central obesity measurements and indices can be applied as additional information to BMI in order to stratify the risks of GDM, as well as other obesity-related conditions and metabolic syndrome.

As the study was the first in Thailand to evaluate BFI and the risk of GDM, future large-scale studies are needed to further determine which central obesity measurements and indices, either a single or a combination of measurements, would be most appropriate in the identification of GDM risk. In addition, whether these measurements are appropriate in any specific subgroup, for example, females with different BMI, should be further evaluated. The effect of central obesity on pregnancy outcomes should also be determined.

## Conclusions

In conclusion, females with BFI of >0.5 were significantly more likely to have GDM. BFI and BMI are positively correlated, and the diagnostic ability of BFI and BMI for GDM was comparable. Females with both BFI of >0.5 and BMI of ≥25 kg/m^2^ have an increased risk for GDM. However, the value of BFI in predicting GDM in the future is still needed to be further elucidated.
